# How Individual Differences in Empathy Predict Moments of Empathy in Everyday Life

**DOI:** 10.1177/01461672251333823

**Published:** 2025-05-28

**Authors:** Gregory J. Depow, Michael Inzlicht

**Affiliations:** 1 University of California San Diego, La Jolla, USA; 2 University of Toronto, ON, Canada

**Keywords:** empathy, experience sampling, trait-state, individual differences, multilevel modeling

## Abstract

Do trait empathy measures predict how people experience empathy in daily life? Despite considerable research on empathy, we know surprisingly little about how trait measures relate to real-world empathic experiences. In this preregistered analysis of 7,343 experience sampling surveys from a near-representative sample of 246 U.S. adults, we map the connections between validated trait empathy measures and state experiences of empathy. Each component of state empathy—including emotion sharing, perspective taking, and compassion—was significantly predicted by theoretically relevant trait measures. However, trait empathy explained limited variance in daily experiences overall, ranging from just 3% for emotion sharing to 15% for perceived empathic efficacy. Adding emotional valence as a predictor improved model fit and variance explained for most state experiences, highlighting the crucial role of context. Our findings validate trait empathy measures while revealing their limitations in predicting real-world experiences.

## Introduction

One of the most distinctive aspects of human social behavior is our capacity for empathy—the ability to understand, share, and care about others’ emotions. Research on empathy has yielded numerous trait measures that aim to capture individual differences in empathic tendencies (Vieten et al., 2024). Yet a crucial question remains largely unexplored: How well do established trait measures predict actual experiences of empathy in daily life? While trait empathy correlates with important outcomes like prosocial behavior and relationship satisfaction (Decety et al., 2016; Kimmes et al., 2014), we know surprisingly little about whether people who score high on empathy measures actually experience more empathy in their day-to-day interactions.

Here, we examine how established trait empathy measures predict experiences of empathy in everyday life, leveraging an existing experience sampling dataset from a large representative sample of U.S. adults (Depow et al., 2021). This approach increases ecological validity (Schreiter et al., 2013) and allows us to examine empathy across a range of social and emotional contexts (Morelli et al., 2014). Our analysis provides the first comprehensive mapping of trait-state relationships in empathy, validating existing measures while revealing their limitations in capturing real-world experiences. This mapping has implications beyond understanding human psychology, as artificial intelligence systems are increasingly designed to simulate empathic responses (Inzlicht et al., 2024).

To address these research questions, we must first have a clear concept of empathy (Hall & Schwartz, 2019). For many decades, researchers have used a wide range of definitions of empathy (Basch, 1983; Batson, 2009; Cuff et al., 2014; Duan & Hill, 1996; Reik, 1948). This plurality of definitions can drive disagreements on the consequences associated with empathy (Bloom, 2017b; Zaki, 2017). Following others (Cuff et al., 2014; Murphy et al., 2022), we define empathy as a multidimensional construct involving emotion sharing (feeling what others feel), perspective taking (seeing things from the others point of view), and compassion (a feeling of warmth care or concern for the other person). Experiencing any of these could be considered a form of empathy.

These components of empathy tend to co-occur in real-life interactions (Depow et al., 2021) and ecologically valid lab tasks (Schurz, 2021), supporting our multidimensional definition (Davis, 1983). However, the dimensions can be differentiated (Lamm et al., 2011; Marsh, 2022; Singer & Klimecki, 2014), and the dimensions are differentially associated with outcomes depending on the context (Weisz & Cikara, 2021), underscoring the need to examine various aspects of empathy rather than a single average empathy construct. The degree to which dimensions of empathy are experienced in a given moment depends on both individual tendencies and situational factors (Zelenski & Larsen, 2000); in particular valence (Fabi et al., 2019) and discrete emotion identity (Olderbak et al., 2014). The extent to which trait measures of empathy predict state experiences of empathy is not clear.

Understanding the connection between trait and state empathy is crucial not only for validating our measurement tools but also for bridging the gap between laboratory research and real-world experience (Lin et al., 2021). This mapping is particularly important given that empathy occurs in diverse contexts (e.g., Ovsyannikova et al., 2025) that may be difficult to capture in standardized measures or laboratory tasks.

### Trait-State Relationships in Psychology

Personality psychologists have long grappled with how traits manifest in everyday behavior (Funder, 2009). A major theoretical breakthrough came with the conceptualization of traits as density distributions of states (Fleeson, 2001)—the idea that traits represent not fixed behaviors but patterns in how people tend to behave across situations. This framework suggests that someone high in a trait should experience corresponding states more than someone low in that trait. For instance, someone high in extraversion may not be outgoing in every situation, but they should display extraverted behaviors more often than their introverted peers when relevant opportunities arise. Further, state deviations from trait averages are not only due to error but are also driven by social-cognitive mechanisms and situational influences (Fleeson & Jayawickreme, 2015).

Constructs may vary along a continuum of being more “state-like,” with considerable variability across situations, or more “trait-like” with considerable stability (Fleeson, 2001). For example, individual differences in mood tend to be highly variable, whereas intelligence and other forms of ability tend to be highly stable (Geiser et al., 2017). Research on Big Five personality traits provides a useful benchmark for expected trait–state relationships. Single-state reports typically correlate with corresponding traits at moderate levels, ranging from *r* = .21 for extraversion, .24 conscientiousness, .32 emotional stability, .34 agreeableness, and *r* = .37 for intellect/openness (Fleeson & Gallagher, 2009). When averaging across multiple state reports, these correlations increase substantially, suggesting that traits better predict behavioral patterns than individual moments. However, these relationships can vary based on characteristics of the situation (Rauthmann et al., 2015). Some situations may constrain behavior so strongly that individual differences have little room to emerge.

The trait–state relationship becomes even more complex for constructs like empathy that depend heavily on the context (Fabi et al., 2019; Stellar & Duong, 2023). Unlike broad personality traits that might manifest in many situations, empathy requires specific triggers—usually others’ emotional expressions or situations. Therefore, the expression of trait empathy may be more constrained by the situation than traits like emotional stability or openness and be more similar to extraversion which likewise often depends on the presence of others.

Moreover, the trait–state relationship may differ across various components of empathy. Some aspects, like empathic efficacy (perceived confidence and reverse-scored difficulty engaging with others’ emotions), might function more like traditional personality traits with relatively stable manifestation across situations. Others, like emotion sharing, might be more state-like and variable. Understanding these patterns can help clarify what exactly our trait measures capture. We next review seven aspects of state empathy which we predicted would be associated with theoretically relevant trait measures.

### Components of Empathy

The first step to experiencing state empathy is recognizing an *opportunity to empathize*. Empathy is a motivated construct (Ferguson et al., 2020; Zaki, 2014). Thus, while opportunities are dependent on the presence of cues in the environment, they may also be influenced by trait empathy through situation selection and modification processes (Ferguson et al., 2020; Grynberg & López-Pérez, 2018; Thompson et al., 2019). Once an opportunity has been noticed, participants may *decide to empathize or not*, and empathy may occur to a greater or lesser *extent*. Empathy may also vary in terms of which components occur.

*Emotion sharing*, or feeling what others feel, is a key component of empathy. Indeed, some researchers have defined empathy more specifically as sharing the emotions of another person while maintaining a self-other distinction (Bloom, 2017a; Mehrabian & Epstein, 1972; Preston, 2007). This capacity builds on but transcends emotion contagion (Doherty, 1997; Elfenbein, 2014; Preston, 2007), a more primitive process of feeling what others feel without necessarily maintaining a self-other distinction which is observed even in infants who cry upon hearing other babies’ distress.

Long considered an important aspect of empathy (Davis, 1983; Dymond, 1949; Hogan, 1969), *perspective taking* involves the active attempt to understand others’ thoughts and feelings from their point of view. This capacity goes beyond merely recognizing that others have different mental states (i.e., theory of mind); it requires imaginatively adopting their perspective to grasp how they see and experience the world (Dvash & Shamay-Tsoory, 2014).

*Compassion*—a feeling of warmth, care, or concern for another person—adds a crucial motivational dimension to empathy (Goetz et al., 2010). While compassion classically “arises in witnessing another’s suffering” (Goetz et al., 2010, p. 351), it is also reported in response to positive emotional states (Depow et al., 2021, 2025).

A related but distinct phenomenon is *personal distress*—a self-focused feeling of stress or anxiety in the presence of other people’s suffering or negative emotions (Batson et al., 1987; Davis, 1983; Singer & Klimecki, 2014). Often linked with empathy (e.g., Davis, 1983), personal distress is fundamentally different in that it is self-focused rather than other-focused. When chronic, personal distress can lead to burnout and compassion fatigue (Gleichgerrcht & Decety, 2013).

At different times, people may feel more or less *empathic efficacy*, varying in how confident they are about the accuracy of their empathy, and the extent to which they found empathy difficult. Perceptions of efficacy drive willingness to empathize (Cameron et al., 2019; Scheffer et al., 2022) and are impactful to empathizer well-being (Depow et al., 2021). Thus, empathic efficacy is an important aspect of empathy.

### Context and Situational Influences

The experience of empathy depends crucially on context (Stellar & Duong, 2023). Laboratory studies often use standardized stimuli—typically negative emotions like sadness or pain—to elicit empathic responses (Morelli et al., 2015). However, everyday empathy occurs in response to a much broader range of emotions and situations. Two aspects of context are particularly important: the valence (positive vs. negative) of the target emotion (Fabi et al., 2019) and the specific discrete emotion involved (Olderbak et al., 2014).

Most trait measures of empathy were developed with a focus on responses to others’ suffering or distress. However, people report opportunities to empathize with others’ positive emotions about three times as often as opportunities to empathize with negative emotions (Depow et al., 2021). Trait measures developed primarily around negative emotions may not predict empathy for positive experiences. Indeed, recent work suggests that positive and negative empathy may be partially dissociable, especially for emotion sharing (Andreychik & Migliaccio, 2015; Löchner et al., 2022; Murphy et al., 2018), raising questions about whether trait measures capture both capacities equally well.

Just as different discrete emotions convey different social information (Van Kleef, 2009), they may also elicit distinct patterns of empathic response (Stellar et al., 2019). For example, emotional pain such as sadness is more likely to elicit compassion, whereas physical pain is more likely to elicit personal distress (Stellar et al., 2019). Further, empathizing with someone’s sadness likely involves different psychological processes than empathizing with their anger or fear, and these differences could manifest in varying relationships between traits and states.

In sum, contextual factors such as valence and discrete emotion may influence the experience of state empathy. Further, they may moderate the relationship between traits and states. For example, measures of positive emotion sharing may only predict state experiences of sharing positively valenced emotions, such as happiness or humor. Understanding the main effects and moderating effects of valence and discrete emotion is thus crucial for improving trait measures and developing more nuanced theories of how empathic dispositions manifest in daily life.

### Current Study

We examine how trait empathy measures predict daily experiences of empathy using experience sampling methodology with a large, representative sample of U.S. adults. We address two preregistered hypotheses: (1) trait empathy measures will be predictive of state empathy in daily life, and specifically (2) selecting empathy via the “feel deck” on the Empathy Selection Task more often will predict noticing more opportunities to empathize in daily life. To address these hypotheses, we map connections between established trait measures (e.g., Empathy Selection Task) and tasks and various aspects of state empathy (e.g., everyday empathy opportunities). Our approach allows us to examine whether trait measures predict state experiences, whether state experiences are predicted by valence and discrete emotion, and how trait–state relationships vary across different components of empathy and different emotional contexts.

Based on the trait–state framework discussed above, we expect significant correlations between trait measures and theoretically related states, such as trait empathic concern predicting state compassion, and trait perspective taking predicting state perspective taking.

We explore four key research questions, each addressing a crucial aspect of trait–state relationships in empathy:

1. Which trait measures best predict specific state experiences? This analysis examines whether theoretically related traits and states show stronger connections (e.g., trait perspective taking predicting state perspective taking) and identifies which aspects of state empathy are best captured by current trait measures.
2. How much variance in daily empathy is explained by trait measures overall? By examining full models with multiple trait predictors, we can assess how well our current measurement tools capture real-world empathic experiences.
3. In comparison, how much variance in state empathy is explained by the context? By comparing the predictive power of trait empathy and situation variables (valence, discrete emotion), we can test whether different dimensions of empathy are more trait-like or state-like.
4. How do emotional context variables (valence and discrete emotion) influence trait-state relationships? This analysis examines whether trait–state relationships vary systematically across different types of emotional situations, providing insight into the generalizability of trait measures.

This comprehensive examination of trait–state relationships in empathy has important implications for both measurement and theory. Beyond validating existing measures, our results can guide the development of more precise tools for capturing individual differences in empathy. Moreover, understanding how traits manifest in daily experience can inform interventions aimed at cultivating empathy and preventing adverse outcomes like burnout.

## Materials and Methods

### Procedure

The data for this study were collected by Depow et al. (2021) to describe empathy in everyday life and examine connections with well-being and prosocial behavior. This rich dataset was previously leveraged to look at how “early birds” and “night owls” vary in their well-being, empathy, and prosociality throughout the day (Francis et al., 2021), and to examine how prosociality and empathy vary over the adult lifespan (Pollerhoff et al., 2022). Open access to data, materials, and code is provided at: https://osf.io/bskwn.

Here, we test a broad preregistered question of theoretical importance: whether trait empathy predicts momentary states of empathy in daily life (https://osf.io/aeqgn). Quota-sampling in partnership with Qualtrics was used to obtain a sample of 246 U.S. adults which was representative of the population on sex, ethnicity, education, region, income, and age according to census data. This total N allows for stable correlation and parameter estimation (Maxwell et al., 2008; Schönbrodt & Perugini, 2013).

### Trait Empathy Measures

**Figure 1.**
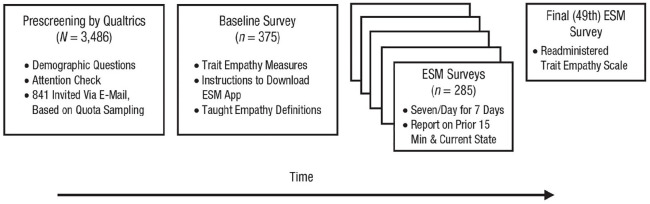
Flow of sampling and data-collection procedure. *Source*. Reproduced with permission from Depow et al. (2021). *Note*. For the experience-sampling method (ESM) surveys, 285 participants completed at least one experience-sampling survey, but 39 participants were excluded for having fewer than seven surveys overall according to our preregistration, leaving a final sample size of 246 to be analyzed.

Trait empathy was measured during the baseline survey and reassessed at the 49th and final experience sampling survey (Figure 1). The most commonly used measure of empathy in the literature is the *Interpersonal Reactivity Index* (Davis, 1983) which contains four subscales: empathic concern (α = .82), perspective taking (α = .78), fantasy (α = .79), and personal distress (α = .81). The empathic concern subscale measures the tendency to feel care or concern for others. We would therefore expect this scale to predict compassion in daily life. Perspective taking measures the tendency to consider the thoughts and viewpoint of others (Dymond, 1949; Hogan, 1969), and would be expected to predict state perspective taking. The fantasy subscale measures how inclined individuals are to become caught up in the feelings of fictional characters and may be expected to predict opportunities to empathize at the state level. Finally, the personal distress subscale taps the tendency to feel distressed when faced with the suffering of others and would be expected to predict state personal distress.

None of the Interpersonal Reactivity Index subscales tap emotion sharing, which is central to empathy (e.g., Mehrabian & Epstein, 1972; Preston & de Waal, 2002). Thus, we also used the *Empathy Index*, which contains an empathy subscale (α = .74) that measures the tendency to share emotions, and a behavioral contagion subscale (α = .72) which measures the tendency to share physical sensations such as itches and yawns (Jordan et al., 2016). We use an empirically derived alternative factor solution for the Empathy Index (Murphy et al., 2018) which groups the items by valence into distress contagion (α = .61), and positive and neutral contagion (α = .67) to test whether trait emotion sharing measures are valence-specific. Theoretically, trait measures of emotion sharing such as the Empathy Index (Jordan et al., 2016) should be the best predictor of state emotion sharing. However, emotion sharing may differ by valence, with individuals differentially sharing positive versus negative emotions (Andreychik & Migliaccio, 2015; Löchner et al., 2022; Murphy et al., 2018). Thus, we may expect valence-specific subscales of the Empathy Index (Murphy et al., 2018), to predict positive and negative emotion sharing, respectively.

As a behavioral measure of willingness to empathize, we used the *Empathy Selection Task*, which requires participants to choose between a “describe” deck and a “feel” deck (Ferguson & Inzlicht, 2023). They are then presented with a face expressing an emotion and must either remain objective and describe the facial expression or feel what the person pictured is feeling and describe the internal emotion. Participants completed 30 trials. Typically, the feel deck is rated as more effortful and avoided. However, this avoidance can be removed by manipulating perceptions of efficacy on the task (Cameron et al., 2019). We preregistered the hypothesis that selecting the feel deck more often on this task would be associated with reporting more opportunities to empathize in daily life.

We administered the *Single Item Trait Empathy Scale* which asks participants directly how much they agree with the statement “I am an empathetic person” (Konrath et al., 2018). This is the only trait measure in the battery which actually uses the word “empathy.” Associations between the Single Item Trait Empathy Scale and state empathy experiences can serve as a window into lay perceptions of empathy (Hall et al, 2020). Our final measure of trait empathy was the *Beliefs about Malleability of Empathy Scale* (α = .89) which measures the extent to which participants believe empathy can be changed with practice (Schumann et al., 2014). As lay definitions of empathy, like researcher definitions, vary widely (Hall et al., 2020), we defined empathy for participants in the baseline survey (see Appendix B Baseline Survey: https://osf.io/bskwn/).

### State Empathy Measures

Following the baseline survey, we used experience sampling (Shiffman et al., 2008) to capture state empathy in everyday life. We used the MetricWire app to prompt participants 7 times a day for 1 week and ask whether they had an *opportunity to empathize* in the last 15 min. When participants indicated an opportunity, they were asked about the *valence* of the emotion observed, the closeness of the person expressing it, how much *personal distress* they felt at the time, and whether they actually *experienced empathy* for the other person. If empathy was reported, participants reported whether they engaged in *emotion sharing*, *perspective taking*, and *compassion*, respectively. For each component, participants rated extent, difficulty, and confidence (for full items see Supplemental Material). Confidence and difficulty of all components were combined and averaged (with difficulty reverse scored) to assess *empathic efficacy* (α = .76).

**Figure 2.**
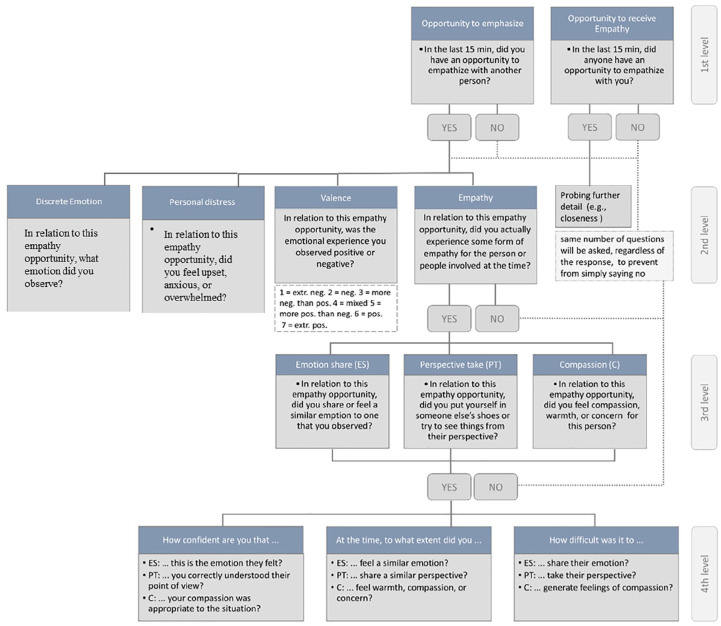
Daily survey design, visualizing the different survey levels and state empathy-related questions. *Source*. Figure modified from Pollerhoff et al. (2022). *Note*. Illustrates how state empathy was assessed in experience sampling surveys. *Empathy opportunities* were assessed first (opportunities to empathize). Given an opportunity, context information (*valence* and *discrete emotion*) was collected, and state *personal distress* and *decisions to empathize* (Empathy Y/N) were assessed. When empathy occurred, each component was assessed (emotion sharing, perspective taking, compassion), and for each component reported, extent, confidence, and difficulty was assessed. Extent ratings were combined with binomial questions such that scores ranged from 0 to 7, where 0 means No (extent of *emotion sharing*, *perspective taking*, and *compassion*). Extent ratings from all three were combined to assess *extent of empathy*. Confidence and difficulty ratings for all three components were averaged and combined into *empathic efficacy*. Only the questions, relevant for the current study, are depicted here. For further details and a full study protocol, see Depow et al. (2021).

For extent of emotion sharing, compassion, and perspective taking, we combined binomial questions (yes/no) with extent ratings (1–7) to create a variable ranging from 0 to 7, where 0 indicates “not at all,” 1 indicates “very little,” and 7 indicates “very much.” In line with our tripartite definition, extent ratings of all three components were combined (α = .72) to assess *extent of empathy* (Figure 2). If participants did not have an opportunity to empathize, they were asked and effort-matched filler questions to ensure that survey length remained constant. Our experience sampling approach allows us to examine empathy out of the lab in the context of daily life, and to avoid generalizing from specific stimuli (Yarkoni, 2022).

### Situational Factors

When measuring state empathy, we also collected data about the context because, like other traits, situational factors play an important role in the experience of empathy (Fabi et al., 2019; Stellar & Duong, 2023). In particular, valence has been shown to impact empathy, and especially emotion sharing (Löchner et al., 2022; Murphy et al., 2018). Empathy may also vary depending on the specific discrete emotion observed (Olderbak et al., 2014; Stellar et al., 2019). We therefore collected information about the valence of the observed emotion (1: *extremely negative* to 7: *extremely positive* with mixed as the midpoint), and the specific discrete emotion (anger, sadness, happiness, etc.) observed.

## Analysis

We preregistered the hypothesis that higher scores on the Empathy Selection Task (i.e., selecting the feel deck more often) would be associated with more empathy opportunities in everyday life. We also preregistered the hypothesis that trait empathy would be predictive of empathy experiences in daily life (https://osf.io/aeqgn). This latter hypothesis leaves a lot of room for flexibility given the number of trait and state empathy components we have in the current study. We therefore took a systematic four-step approach to address our research questions: (a) single predictor models (to find the best trait predictors of state experiences), (b) full trait empathy models (to estimate variance explained in state experiences by trait empathy overall), (c) situational models with features of the emotional context in isolation, then alongside trait empathy as main effects (to compare the effect of context variables and trait empathy on state empathy), and (d) models testing for interactions between trait empathy and situational variables (to test whether the context moderates trait–state connections).

We used generalized and linear mixed-effects models with grand-mean centered trait empathy measures as fixed-effect predictors. To account for the nested nature of the data, we included random intercepts for participant, survey day, and participant by day combinations. For continuous measures a beta of 1 implies scoring 1 point higher on the predictor is associated with a one-unit change in the dependent variable, while for binomial (yes/no) measures, a beta score of 1 indicates that a change of 1 in the predictor is associated with a change of 1 in the log odds of a yes response on the dependent variable.

Linear mixed-effects models were fit using restricted maximum likelihood (Corbeil & Searle, 1976), whereas generalized mixed-effects models were fit using the maximum likelihood Laplace Approximation (Wolfinger, 1993). Models were constructed in R, using the “lmer” and “glmer” functions from the lme4 package (Bates et al., 2015), and using the lmerTest package (Kuznetsova et al., 2017) to calculate *p*-values. We applied a control using the “bobyqa” optimizer (Powell, 2009). If models did not converge or were singular, we dropped nesting within survey day. If a model was singular or would not converge at this point, it was not interpreted.

The first step involved single-predictor multilevel models to estimate one-to-one relationships between trait empathy measures and state empathy experiences. We predicted each state empathy experience from each trait empathy measure in turn, then adjusted *p*-values for multiple comparisons following the False Discovery Rate procedure (Benjamini & Hochberg, 2007). In this way, we can determine which trait measures best predict which state experiences.

In our second step, we determine how much variance in daily empathy experiences is explained by trait empathy overall using full models. We predicted each aspect of state empathy in daily life in turn using all trait empathy measures in a single model.^1^ Examining the marginal *R*^2^ of these “full models” allows us to determine how much of the variance in each state empathy experience is explained by trait empathy.

### Accounting for Context

In our third step, to understand the impact of the emotional context (Fabi et al., 2019; Stellar & Duong, 2023), we tested models predicting each component of state empathy from valence and discrete emotion alone. Next, we tested full models with emotional valence and discrete emotion included as a main effect with all trait empathy predictors, to see how much additional variance in state empathy experiences is explained.

In a fourth and final step, we tested whether context variables moderated the connection between trait and state empathy by testing for interactions between all trait empathy predictors and context variables. A considerable increase in variance explained and better model fit would indicate that the connection between trait and state empathy varies importantly across emotional contexts.

Valence and discrete emotion variables were coded as factors with negative and pain as reference points, respectively. Thus, we test if experiences differ appreciably when the emotional context varies from the typical stimuli used in empathy research (Morelli et al., 2015).

### Model Comparison

For each state empathy experience, we used model comparison based on Bayesian Information Criterion (BIC) to determine the best model (Schwarz, 1978). We compared models that included all trait empathy predictors (full models), valence alone (valence only), discrete emotion alone (emotion only), trait empathy and valence as a main effect (valence main), and trait empathy and discrete emotion as a main effect (emotion main). We also compared models where valence interacted with trait empathy (valence interaction), and models where discrete emotion interacted with trait empathy (emotion interaction), see Table S2. Winning models give us a sense of the best way to predict state empathy experiences: from trait measures, the context, or some combination of the two.

## Results

We present results from single predictor models to map which trait measures best predict which state experiences. We examine the marginal *R*^2^ of full empathy models for each state experience to determine how much variance in state empathy is explained by trait empathy overall. Finally, we consider the influence of the context.

Results are presented largely in line with the order in which participants answered questions about state empathy. First, we present results for empathy opportunities and engaging in empathy given the opportunity. Second, we present results for the extent to which empathy is experienced (emotion sharing, perspective taking, and compassion combined into a single variable). Third, we present results for each of the three components of empathy in isolation. Fourth, we present results for personal distress during empathy opportunities, followed finally by perceptions of empathic efficacy during experiences of empathy. Following single predictor and full trait empathy model results for each state empathy experience, we present results regarding the influence of the context, both as a main effect, and as a moderator of trait–state relationships.

### Empathy Opportunities and Experiences

The strongest predictor of empathy opportunities^2^ was the Fantasy subscale of the Interpersonal Reactivity Index (Davis, 1983), *b* = 0.39, *SE* = 0.11, *z* = 3.61, adj. *p* = .003, *r* = .11, followed by perspective taking, *b* = 0.36, *SE* = 0.13, *z* = 2.76, adj. *p* = .027, *r* = .10, Table 1. We note these effects are small, around the 25th percentile among effects reported in the social psychology literature (Lovakov & Agadullina, 2021). As hypothesized, the Empathy Selection Task (Cameron et al., 2019), *b* = 0.16, *SE* = 0.07, *z* = 2.32, adj. *p* < .05, *r* = .04, predicted noticing opportunities to empathize in everyday life. Furthermore, believing empathy to be malleable (Schumann et al., 2014), *b* = 0.14, *SE* = 0.06, *z* = 2.30, adj. *p* < .05, *r* = .04, was associated with more empathy opportunities. Both of these effects were very small.

**Table 1.** State and Trait Empathy Connections.

State empathy measures	Perspective taking (IRI)	Empathic concern (IRI),	Personal distress (IRI)	Fantasy (IRI)	Positive/neutral contagion (EI)	Distress contagion (EI)	Empathy selection task	Single item trait empathy scale	Malleability of empathy
Empathy opportunity	0.36*, (0.13), *r* = **.10**	0.09, (0.13), *r* = .03	−0.08, (0.11), *r* = .02	0.39**, (0.11), *r* = **.11**	0.05, (0.11), *r* = .01	−0.21, (0.10), *r* = .06	0.16*, (0.07), *r* = **.04**	0.14, (0.10), *r* = .04	0.14*, (0.06), *r* = **.04**
Decision to empathize	0.56, (0.25), *r* = .15	1.13***, (0.22), *r* = **.30**	−0.05, (0.22), *r* = .02	0.43, (0.21), *r* = .12	0.36, (0.21), *r* = .10	0.11, (0.20), *r* = .03	0.11, (0.14), *r* = .03	0.62***, (0.18), *r* = **.17**	0.26, (0.12), *r* = .07
Extent of empathy	0.20*, (0.08), *r* = **.19**	0.31***, (0.08), *r* = **.28**	−0.05, (0.07), *r* = .06	0.11, (0.07), *r* = .13	0.19*, (0.07), *r* = **.20**	0.007, (0.06), *r* = .01	0.08, (0.04), *r* = .12	0.26***, (0.06), *r* = **.31**	0.04, (0.04), *r* = .05
Emotion sharing	0.43*, (0.15), *r* = **.22**	0.44*, (0.15), *r* = **.21**	0.09, (0.13), *r* = .05	0.24, (0.13), *r* = .14	0.32*, (0.13), *r* = **.19**	0.09, (0.12), *r* = .06	0.06, (0.08), *r* = .05	0.29*, (0.12), *r* = **.18**	0.05, (0.07), *r* = .06
Compassion	0.34*, (0.12), *r* = **.22**	0.60***, (0.12), *r* = **.37**	−0.05, (0.11), *r* = .04	0.16, (0.11), *r* = .12	0.20, (0.11), *r* = .15	0.07, (0.10), *r* = .06	0.16, (0.07), *r* = .17	0.46***, (0.09), *r* = **.36**	0.09, (0.06), *r* = .12
Perspective taking	0.57**, (0.16), ***r*** = **.26**	0.54**, (0.16), *r* = **.23**	−0.08, (0.14), *r* = .04	0.38*, (0.14), *r* = **.20**	0.22, (0.14), *r* = .12	0.07, (0.13), *r* = .04	0.07, (0.09), *r* = .06	0.30*, (0.13), *r* = **.17**	0.05, (0.08), *r* = .05
Personal distress	−0.21, (0.13), *r* = .12	−0.05, (0.12), *r* = .03	0.20, (0.11), *r* = .12	−0.08, (0.11), *r* = .05	−0.007, (0.11), *r* = .01	0.35***, (0.10), *r* = **.25**	0.05, (0.07), *r* = .05	−0.03, (0.10), *r* = .02	−0.11, (0.06), *r* = .13
Empathy efficacy	0.27***, (0.07), *r* = **.27**	0.40***, (0.07), *r* = **.38**	−0.20***, (0.06), *r* = **.22**	0.04, (0.06), *r* = .05	0.17*, (0.06), *r* = **.19**	−0.09, (0.06), *r* = .11	0.02, (0.04), *r* = .03	0.18**, (0.06), *r* = **.22**	0.05, (0.04), *r* = .11

*Note*. Rows are state empathy, columns are trait empathy. Individual cells have *b* coefficients, standard errors, and *r* values from single predictor models. For example, a one-unit change in trait perspective taking is associated with a change of *b* = 0.57 in state perspective taking, corresponding to an effect size of *r* = .26. Statistics flagged for significance according to False Discovery Rate adjusted *p*-values, where *p < .05, **p < .01, ***p < .001. Statistically significant values are bolded. We calculate *r* values using a validated effect size for multilevel models derived from *R*^2^ (Edwards et al., 2008). Big Five trait-single state correlations for comparison: *r* = .21 for extraversion, .24 conscientiousness, .32 emotional stability, .34 agreeableness, and *r* = .37 for intellect (Fleeson & Gallagher, 2009).

The marginal *R*^2^ of our full-trait empathy model suggested trait empathy explained 7% of the variance in reported empathy opportunities in the last 15 min. While 7% may seem low, it is difficult to determine how much of the variance in empathy opportunities, we would expect to be explained by trait empathy,^3^ because whether an empathy opportunity is present or not also depends on the environment.

Given an opportunity, full-trait empathy models explained about 13% of the variance in decisions to empathize (Table 2). Empathic Concern from the Interpersonal Reactivity Index showed a medium effect, a bit over the 50th percentile (Lovakov & Agadullina, 2021), of predicting whether people decided to empathize, *b* = 1.13, *SE* = 0.22, *z* = 5.06, adj. *p* < .001, *r* = .30. In addition, the Single-Item Trait Empathy Scale showed a small effect, *b* = 0.61, *SE* = 0.18, *z* = 3.47, adj. *p* = .003, *r* = .17. No other trait predictors in the model emerged as significant (all *p*’s > .05).

**Table 2.** Variance Explained by Trait Empathy and Context Variables.

State empathy	Trait empathy	Valence	Emotion
Empathy efficacy	*R*^2^ = 15%	*R*^2^ = 3% (**18%**) 19%	*R*^2^ = 3% (17%) 22%
Decision to empathize	*R*^2^ = **13%**	*R*^2^ = <1% (13%) 18%	*R*^2^ = 5% (17%) —
Compassion	*R*^2^ = 7%	*R*^2^ = 1% (**8%**) 9%	*R*^2^ = 3% (10%) 17%
Personal distress	*R*^2^ = 7%	*R*^2^ = 27% (**33%**) 33%	*R*^2^ = 28% (33%) 39%
Extent of empathy	*R*^2^ = **7%**	*R*^2^ = 1% (8%) 10%	*R*^2^ = 3% (10%) 17%
Perspective taking	*R*^2^ = 4%	*R*^2^ = <1% (**4%**) 5%	*R*^2^ = 1% (5%) 14%
Emotion sharing	*R*^2^ = 3%	*R*^2^ = 4% **(7%)** 11%	*R*^2^ = 6% (9%) 18%

*Note*. Marginal variance explained by different models. The first column shows the marginal *R*^2^ for full trait empathy models, indicating the amount of variance in a given state empathy experience explained by trait empathy measures overall. The second column illustrates the variance explained by valence. The first number is valence alone, the second number in parentheses is with both valence and all trait empathy predictors as main effects, and the third number is variance explained when testing for interactions between valence and all trait empathy predictors. The final column illustrates the same with specific discrete emotion rather than valence. The model for empathy decisions which tests for interactions between discrete emotion and trait empathy predictors would not converge and so is not included (marked with—). In each row, the number that is bolded indicates the winning model based on BIC comparison. In 5 out of 7 state empathy experiences, the best model involved trait empathy plus valence as a main effect. The remaining 2 winning models were trait empathy variables without context information.

### Extent of Empathy

We combined emotion sharing, perspective taking, and compassion extent ratings as a measure of the extent to which individuals experienced empathy. Single predictor models showed that the single item trait empathy scale, *b* = 0.26, *SE* = 0.06, *t*(186) = 4.49, adj. *p* < .001, *r* = .31, as well as empathic concern, *b* = 0.31, *SE* = 0.08, *t*(196) = 4.13, adj. *p* < .001, *r* = .28, perspective taking, *b* = 0.20, *SE* = 0.08, *t*(179) = 2.64, adj. *p* = .020, *r* = .19, and positive/neutral contagion, *b* = 0.19, *SE* = 0.07, *t*(181) = 2.80, adj. *p* = .020, *r* = .20, were all positively associated with extent of empathy in everyday life. Our full-trait empathy model explained 7% of the variance in extent of empathy overall.

### Emotion Sharing

Four trait empathy predictors were significantly associated with emotion sharing in everyday life: empathic concern, *b* = 0.44, *SE* = 0.15, *t*(179) = 2.94, adj. *p* = .018, *r* = .21, perspective taking, *b* = 0.43, *SE* = 0.15, *t*(168) = 2.91, adj. *p* = .018, *r* = .22, the Single Item Trait Empathy Scale, *b* = 0.29, *SE* = 0.12, *t*(172) = 2.44, adj. *p* = .034, *r* = .18, and the positive neutral contagion subscale of the Empathy Index, *b* = 0.32, *SE* = 0.13, *t*(163) = 2.48, adj. *p* = .034, *r* = .19. The distress contagion subscale, *b* = 0.09, *SE* = 0.12, *t*(153) = 0.74, adj. *p* = .512, *r* = .06, which taps negative trait emotion sharing, was not associated with overall emotion sharing in daily life. However, distress contagion did predict sharing *negative* emotions at the state level, *b* = 0.65, *SE* = 0.23, *t*(86) = 2.83, *p* = .006, *r* = .29, underscoring the potentially valence specific nature of trait emotion sharing. Our full trait empathy model explained only 3% of the variance in emotion sharing. This is a notably small portion of the variance, suggesting trait empathy measures used in this study may not adequately capture emotion sharing.

### Compassion

Three trait empathy measures were significant predictors of experiences of compassion in everyday life. As we might expect, foremost among them was empathic concern, *b* = 0.60, *SE* = 0.12, *t*(161) = 5.00, adj. *p* < .001, *r* = .37. Interestingly, individuals who thought of themselves as “an empathetic person” as per the Single-Item Trait Empathy Scale, reported feeling more compassion in everyday life, *b* = 0.45, *SE* = 0.09, *t*(155) = 4.85, adj. *p* < .001, *r* = .36. In addition trait perspective taking was associated with increased compassion, *b* = 0.34, *SE* = 0.12, *t*(153) = 2.73, adj. *p* = .018, *r* = .22, though the effect was smaller. Overall, results from our full model showed that trait empathy explained around 7% of the variance in state experiences of compassion in everyday life.

### Perspective Taking

We found four significant trait predictors of momentary perspective taking in everyday life. As theoretically expected, the strongest predictor of state perspective taking was the perspective taking subscale of the Interpersonal Reactivity Index, *b* = 0.57, *SE* = 0.16, *t*(190) = 3.63, *p* < .001, *r* = .26. Trait empathic concern, *b* = 0.54, *SE* = 0.16, *t*(201) = 3.35, adj. *p* = .003, *r* = .23, and fantasy, *b* = 0.38, *SE* = 0.14, *t*(182) = 2.78, adj. *p* = .018, *r* = .20, were also associated with increased everyday perspective taking. Finally, the single-item trait empathy scale was associated with increased perspective taking in daily life, *b* = 0.30, *SE* = 0.13, *t*(189) = 2.38, adj. *p* = .040, *r* = .17. Thus, state perspective taking was predicted by multiple trait indicators, underscoring the overlapping nature of these constructs (Depow et al., 2021). Overall, trait empathy explained 4% of the variance in perspective taking in everyday life.

### Personal Distress

Single-predictor models revealed just one significant trait empathy predictor of everyday personal distress: the distress contagion scale, *b* = 0.35, *SE* = 0.10, *t*(185) = 3.54, adj. *p* = .005, *r* = .25, which indexes the tendency to share negative emotions. Somewhat surprisingly, trait personal distress was not associated with increased personal distress in daily life, *b* = 0.20, *SE* = 0.11, *t*(214) = 1.78, adj. *p* = .230, *r* = .12. Positive/neutral contagion did not predict state personal distress, *b* = −0.007, *SE* = 0.11, *t*(193) = −0.06, *p* = .950, *r* = .004, suggesting that while trait emotion sharing can lead to distress, this is specific to the tendency to share negative emotions. No other trait empathy measures were significantly associated with personal distress in everyday life (all *p*’s > .05). Results from our full trait empathy model show trait empathy explains about 7% of the variance in personal distress overall.

### Empathic Efficacy

Individuals who scored high in empathic concern reported greater empathy efficacy at the state level, *b* = 0.40, *SE* = 0.07, *t*(192) = 5.69, adj. *p* < .001, *r* = .38. Similarly, perspective taking, *b* = 0.27, *SE* = 0.07, *t*(181) = 3.73, adj. *p* = .001, *r* = .27, the single-item trait empathy scale, *b* = 0.18, *SE* = 0.06, *t*(185) = 3.10, adj. *p* = .004, *r* = .22, and positive/neutral contagion, *b* = 0.17, *SE* = 0.06, *t*(182) = 2.67, adj. *p* = .014, *r* = .19, predicted empathy efficacy. Trait personal distress was associated with reduced perceptions of empathy efficacy in everyday life, *b* = −0.20, *SE* = 0.06, *t*(194) = −3.08, adj. *p* = .005, *r* = .22. Despite the fact that none of the trait measures explicitly aim to capture perceptions of empathic efficacy, trait empathy explained 15% of the variance in efficacy overall. Thus, trait empathy captured perceptions of efficacy during empathy experiences better than any other aspect of state empathy.

### Considering the Context: Valence and Emotion as Main Effects and Moderators

Valence played an important role in state empathy. Models predicting state empathy using trait empathy and valence as a main effect were selected as the best model for 5 out of 7 state empathy experiences: efficacy, compassion, personal distress, emotion sharing, and perspective taking. The remaining two state experiences—empathizing or not (given an opportunity) and extent of empathy experienced—were best explained by trait empathy alone (Table 2). Different aspects of empathy were differentially impacted by the emotional context. Less than 1% of the variance in state perspective taking was explained by valence, but valence explained 4% of the variance in state emotion sharing—more than trait empathy—suggesting that emotion sharing may be more “state-like” than perspective taking. A massive 27% of the variance in personal distress was explained by valence, underscoring the substantial influence of valence on feelings of distress. Knowing more specifically which emotion participants observed did not improve the model for any aspect of state empathy, suggesting valence itself plays a key role in shaping state empathy experiences.

Participants reported greater empathy efficacy, *b* = 0.41, *SE* = 0.06, *t*(1108) = 7.12, *p* < .001, *r* = .21, emotion sharing, *b* = 0.88, *SE* = 0.16, *t*(1129) = 5.51, *p* < .001, *r* = .16, and compassion, *b* = 0.28, *SE* = 0.12, *t*(1122) = 2.21, *p* = .027, *r* = .07, as well as much less personal distress, *b* = −2.44, *SE* = 0.11, *t*(1294) = −22.54, *p* < .001, *r* = .53, when engaging with positive emotions relative to negative emotions.

They also felt less distress, *b* = −0.94, *SE* = 0.14, *t*(1285) = −6.67, *p* < .001, *r* = .18, and shared emotions to a greater extent, *b* = 0.92, *SE* = 0.18, *t*(1123) = 5.04, *p* < .001, *r* = .15, during mixed emotions relative to negative. Interestingly, perceptions of empathy efficacy during mixed emotions did not differ significantly from efficacy during negative emotions, *b* = 0.12, *SE* = 0.07, *t*(1084) = 1.62, *p* = .105, *r* = .05. Testing for interactions between trait empathy and features of the context did not result in better models, suggesting that while the situation influenced experiences of state empathy as a main effect, it did not moderate trait-state connections.

## General Discussion

Our findings illuminate a fundamental tension in how empathy operates: individuals show stable tendencies in their empathic responses, which are captured by theoretically predictable trait measures, yet these dispositions explain surprisingly little variance in moment-to-moment empathic experiences. This suggests empathy may be better conceptualized as a dynamic process (Murphy et al., 2022) that individuals deploy differently across contexts, rather than a purely dispositional tendency (Stellar & Duong, 2023). State experiences were often predicted by multiple trait predictors, highlighting the interconnected nature of the empathy components (Depow et al., 2021).

The strong influence of valence on empathy experiences, particularly for emotion sharing and personal distress, suggests that the mechanisms underlying empathy may operate differently for positive versus negative emotions (Andreychik, 2019). This asymmetry could reflect evolutionary adaptations—sharing positive emotions may promote well-being (Depow et al., 2025) and serve affiliative functions (Ringwald & Wright, 2021), while the sharing of negative emotions could have evolved to alert us to threats or provide motivation for helping behavior (Decety, 2011). While perspective taking and compassion were less influenced, the fact that valence alone explained 27% of variance in personal distress (compared to 7% explained by trait measures) indicates that the situational emotional context may be more important than individual differences in shaping certain aspects of empathic experiences (Fabi et al., 2019). This finding has important implications for how we conceptualize and measure empathy.

While comparisons should be made cautiously given different statistical methods were used to yield the effect sizes, it is useful to situate single-predictor trait—single-state empathy relationships within the context of past research on personality predicting single state reports of personality (Fleeson & Gallagher, 2009). Trait empathy predictors of whether or not an opportunity to empathize was reported in daily life showed much smaller effects than personality, with the largest effect (Interpersonal Reactivity Index Fantasy score, *r* = .11) being roughly half the size of extraversion, the smallest personality trait–single-state relationships. However, within the context of an empathy opportunity, we find robust trait–state relationships in empathy that rival those found in personality research. Self-reporting as an empathetic person predicted extent of state empathy as strongly as emotional stability predicts its corresponding states (*r* = .32). Even more impressive, the relationship between trait empathic concern and state compassion matched that of intellect (*r* = .37), which shows the strongest trait–state correspondence among Big Five personality dimensions (see Table 1). Thus, trait empathy predicts shows trait–state relationships comparable to Big Five trait–state relationships in empathy relevant situations (Funder, 2009; Rauthmann et al., 2015).

Comparing trait empathy and Big Five trait–state mean correlations (Fleeson & Gallagher, 2009), we see Big Five relationships tend to be larger, ranging from .38 to .53, than trait-state mean empathy correlations, ranging from .20 to .31 (Table S5). This pattern suggests empathy may function differently from basic personality traits—while people act on their empathic dispositions when situations clearly call for empathy, these tendencies may be more selectively deployed rather than manifesting consistently across all situations. This aligns with theoretical perspectives viewing empathy as a motivated capacity (Zaki, 2014) that people can choose to engage rather than a trait that operates uniformly across contexts.

Full trait empathy models explained more variance than the Big 5 (Soto & John, 2017) for all state empathy experiences aside from personal distress and compassion, which were well-predicted by neuroticism and agreeableness (see Table S1). Nonetheless, many of the theoretically expected trait–state empathy relationships were robust to controlling for other trait empathy measures and Big 5 scores (see Supplemental Material, p. 1), suggesting they predict unique variance which cannot be subsumed within the Big 5 framework (Bainbridge et al., 2022).

Although trait empathy largely predicted theoretically relevant state experiences, some measures did not display expected validity. Trait personal distress (IRI; Davis, 1983) did not predict state personal distress, though it did show a small correlation (*r* = .15, *p* = .02) at the mean level. Additionally, emotion sharing, despite being central to empathy, was poorly predicted by trait empathy measures overall. While several trait measures correlated with state emotion sharing when analyzed individually, the empathy subscale of the Empathy Index (Jordan et al., 2016)—which specifically measures trait emotion sharing—showed no significant relationship (*p* > .05).

The valence and trait empathy interaction model was not the winning model for predicting emotion sharing, yet there is reason to suspect this trait-state link may be valence-specific (Andreychik & Migliaccio, 2015; Löchner et al., 2022). Here, our measure of trait emotion sharing, the Empathy Index (Jordan et al., 2016) predicted emotion sharing only when emotions were negative. The positive neutral contagion subscale (Murphy et al., 2018) predicted emotion sharing overall in this dataset, where positive emotions were more common (Depow et al., 2021), while the distress contagion predicted sharing negative, but not positive, emotions. Only 3% of the variance in everyday emotion sharing was explained by trait empathy. However, if we restrict our analysis to empathy opportunities for negative emotions, 7% of the variance in emotion sharing is explained by trait empathy. This may reflect the focus on negative emotions in our trait empathy items (Morelli et al., 2015). Future work should develop better measures for capturing positive emotion sharing (e.g., Light et al., 2019), and further examine how valence-specific trait emotion sharing measures predict valence-specific state emotion sharing.

### Single Item Trait Empathy Scale as a Window into Lay Perceptions

Lay definitions of empathy are variable and comprised of multiple dimensions (Hall et al., 2020). The single item trait empathy scale, which asks participants to rate their agreement with the statement “I am an empathetic person,” is the only trait-level item which uses the word “empathy” and thus can serve as a window into lay conceptions of empathy.

Individuals who considered themselves empathetic were more likely to choose to empathize given an opportunity. They also shared emotions, took perspective, and felt compassion to a greater extent, and felt more efficacious in their empathy. On the other hand, participants who described themselves as empathetic were no more likely to report opportunities to empathize. They also experienced personal distress at similar levels to those who did not describe themselves as empathetic.

**Figure 3.**
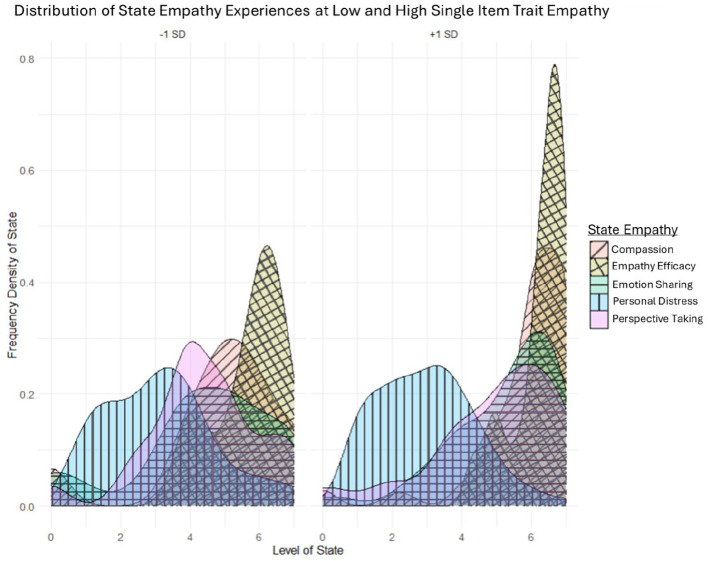
Landscape of state empathy at low and high single item trait empathy scale scores. *Note*. Participants scoring low on the single item trait empathy scale—indicating they did not consider themselves an empathetic person—reported lower perceived efficacy during empathy in everyday life. They were lower on average in perspective taking, emotion sharing, and compassion, and therefore showed a greater overlap between these components of empathy and personal distress. In sum, rating oneself as an empathetic person at the trait level is associated with a shifted landscape of state empathy experience.

While researchers have strongly differentiated empathy and compassion (Singer & Klimecki, 2014), defining empathy specifically as feeling what others feel while maintaining a self-other distinction, individuals who thought of themselves as an empathetic person felt more compassion in everyday life. This observation lends support to findings that compassion is central to lay conceptions of empathy (Hall et al., 2020). Put simply, for lay-people feeling compassion in everyday life is a key component of what it means to be an empathetic person. Participants who reported often taking the point of view of others at the trait level, also reported feeling more compassion in everyday life (Batson, 2019), again highlighting the overlapping nature of empathy. In sum, describing oneself as empathetic at the trait level was associated with a different landscape of state empathy experiences, Figure 3, shifting many but not all aspects of state empathy.

This study has several important strengths including a representative sample, ecologically valid empathy opportunities, and a rich dataset. Nevertheless, it also brings along a number of limitations.

### Limitations and Future Directions

Testing for interactions between discrete emotion and trait empathy allows us to determine whether trait measures predict state experiences similarly across different emotions. However, some emotions were less common in the dataset than others. This limited sample size for less common emotions limits our ability to detect and reliably estimate the size of trait empathy effects. In the future, researchers interested in state empathy during specific emotions may benefit from a dataset which has more observations of their emotions of interest, such as disgust, contempt, and embarrassment.

In the current work, we chose the best model based on BIC comparison. However, BIC favors the simpler model with fewer parameters than alternatives such as the corrected Akaike Information Criterion (AICc) (Burnham & Anderson, 2004). Choosing AICc for model comparison would have led to more complex models being selected as the best model in some cases. Thus, while our winning models involved either trait empathy or trait empathy with valence as a main effect, future researchers may still wish to explore how the connection between trait empathy and state empathy—particularly emotion sharing—may vary across valence or discrete emotion. For BIC, AICc, Inta-class Correlation (ICC), and *R*^2^ of all compared models see Table S3.

Not explored in the current analysis, but also important, is that cues occur in a particular *social* context (Main, 2022). That is, they are elicited by a specific target person, sometimes as a response to other people or the empathizer themselves. The current dataset has closeness of the target, but we focus on features of the emotional context here. Nonetheless, social factors such as closeness and similarity can also influence the empathy experience and should be considered in future research.

Finally, while this study used quota-sampling to ensure the sample was representative of the U.S. population on key demographics, thereby improving generalizability to the larger population of U.S. adults, generalizability to other groups, especially non-WEIRD populations (Henrich et al., 2010), is unclear. These limitations notwithstanding, the current work makes an important contribution to our understanding of how trait empathy measures predict state empathy experiences in everyday life.

### Conclusion

This research advances our understanding of empathy as both a trait and a state-level phenomenon. Our findings reveal empathy as a complex psychological process that operates at the intersection of stable individual differences and situational demands. While trait measures capture meaningful variations in how people typically respond empathically, the modest amount of variance they explain in daily experiences suggests empathy is fundamentally contextual (Stellar & Duong, 2023). The strong influence of emotional valence on certain aspects of empathy (personal distress, emotion sharing) but not others (perspective taking, compassion) suggests that different components of empathy take different places on the trait-like to state-like continuum.

Our findings also highlight important gaps in current measurement approaches. While some aspects of empathy are well-captured by existing trait measures, others—particularly emotion sharing and personal distress—may require new measurement strategies that better account for contextual factors and valence-specific effects (Gamble et al., 2024; Löchner et al., 2022). Understanding these measurement limitations is crucial as researchers continue to investigate how empathy operates in daily life and develop interventions to promote adaptive empathic responses.

## Supplemental Material

sj-docx-1-psp-10.1177_01461672251333823 – Supplemental material for How Individual Differences in Empathy Predict Moments of Empathy in Everyday Life Supplemental material, sj-docx-1-psp-10.1177_01461672251333823 for How Individual Differences in Empathy Predict Moments of Empathy in Everyday Life by Gregory J. Depow and Michael Inzlicht in Personality and Social Psychology Bulletin

## Notes

1. Trait empathy measures are often correlated, and this data are no exception (Figure S1). To ensure we would not have issues with multicollinearity, we calculated the variance inflation factor for each predictor in each model, following a rule of thumb to remove any predictors with a variance inflation factor greater than five (James et al., 2013). However, all predictors had a variance inflation factor below 3 and were therefore retained.

2. When predicting empathy opportunities, we obtained a singular fit when survey day was included as a random intercept. Therefore, we report results nested within participant as a random intercept only. Results are robust to including survey day as a fixed effect, though we do observe a negative effect of survey day, *b* = −0.18, *SE* = 0.02, *z* = −10.05, *p* < .001, *r* = −.05. Further, if we use a linear model to predict mean empathy opportunities of each participant with trait empathy predictors, all findings are robust aside from malleability of empathy.

3. By way of comparison, 2.6% of the variance in empathy opportunities is explained by the Big-5 factor scores. For further comparisons of trait empathy to Big-5 factors in predicting state empathy experiences, see Table S1.
