# Supplementary material for: How Individual Differences in Empathy Predict Moments of Empathy in Everyday Life
Source: Pers Soc Psychol Bull. 2025 May 28;52(8):2529–44. doi: 10.1177/01461672251333823 (PMC13310264; doi:10.1177/01461672251333823)
Supplement: sj-docx-1-psp-10.1177_01461672251333823 – Supplemental material for How Individual Differences in Empathy Predict Moments of Empathy in Everyday Life [file sj-docx-1-psp-10.1177_01461672251333823.docx]

**Supplementary Material**

**for**

**How individual differences in empathy predict moments of empathy in everyday life**

**Table S1.** Comparing Big 5 and Trait Empathy in Explaining State Empathy Variance.

| **Variable** | **Big 5** | **Trait Empathy** |
| --- | --- | --- |
| Empathy Opportunity (Y/N) | 0.026 | **0.065** |
| Empathy Given Opp. (Y/N) | 0.079 | **0.128** |
| Extent of Empathy | 0.066 | **0.067** |
| Emotion Sharing | 0.023 | **0.030** |
| Perspective Taking | 0.032 | **0.037** |
| Compassion | **0.086** | 0.072 |
| Personal Distress | **0.060** | 0.055 |
| Perceived Empathy Efficacy | 0.100 | **0.149** |
| Opp. to Receive Empathy | 0.028 | **0.061** |
| Extent Empathy Received | 0.052 | **0.084** |

*Note.* This table compares variance in state empathy explained by the Big Five personality dimensions and by trait empathy. Trait empathy largely outperformed the Big Five aside from compassion and personal distress. As single predictors, agreeableness predicted compassion, b = 0.81, SE = 0.15, t(152) = 5.53, p < .001, r = 0.41, and neuroticism predicted personal distress, b = 0.46, SE = 0.09, t(215) = 5.34, p < .001, r = 0.34, more strongly than any single trait empathy predictor.

We also examined full models with the Big 5 included, to see whether trait empathy predictors remain significant accounting for trait empathy and a more general measure of personality. In these models, trait empathy measures did not predict extent of empathy, emotion sharing, perspective taking, or compassion in daily life (all p’s > .05). Some relationships were robust, however. Fantasy, b = 0.49, SE = 0.13, z = 3.82, p < .001, r = 0.13, and distress contagion, b = -0.32, SE = 0.14, z = -2.32, p = .020, r = -0.09, predicted empathy opportunities. Empathic concern predicted empathizing given the opportunity, b = 1.11, SE = 0.34, z = 3.22, p = .001, r = 0.29. Distress contagion predicted experiencing personal distress, b = 0.40, SE = 0.14, t(195) = 2.96, p = .004, r = 0.21. Fantasy, b = 0.40, SE = 0.17, z = 2.39, p = .017, r = 0.11, distress contagion, b = -0.48, SE = 0.18, z = -2.64, p = .008, r = -0.13, the SITES, b = 0.34, SE = 0.16, z = 2.16, p = .031, r = 0.09, and the empathy selection task, b = 0.18, SE = 0.09, z = 2.04, p = .041, r = 0.05, predicted opportunities to receive empathy. Empathic concern, b = 0.38, SE = 0.13, t(135) = 2.86, p = .005, r = 0.24, and distress contagion, b = -0.20, SE = 0.10, t(146) = -2.01, p = .046, r = 0.16, correlated with the extent to which empathy was received. These represent robust relationships, where specific trait empathy measures seem to explain unique variance in state empathy experiences, controlling for other trait empathy measures as well as for scores on the Big-5 personality domains.

**Table S2.** BIC for model comparisons

| State Empathy Experience | Trait Empathy | Valence & Empathy | Emotion & Empathy | Valence  Alone | Emotion Alone |
| --- | --- | --- | --- | --- | --- |
| Empathy Efficacy | 2,679 | **(2,586)** 2,688 | (2,696) 3,259 | 2,685 | 2,790 |
| Compassion | 4,438 | **(4,411)** 4,516 | (4,429) 4,994 | 4,541 | 4,596 |
| Personal Distress | 5,320 | **(4,874)** 4,968 | (4,899) 5,452 | 5,053 | 5,127 |
| Extent of Empathy | **4,093** | (4,095) 4,178 | (4,119) 4,661 | 4,219 | 4,304 |
| Perspective Taking | 5,290 | **(5,226)** 5,320 | (5,271) 5,826 | 5,132 | 5,509 |
| Emotion Sharing | 5,084 | **(4,972)** 5,042 | (4,995) 5,548 | 5,132 | 5,215 |

*Note.* The above table shows the BIC associated with each model predicting each state empathy experience. All models are nested include random intercepts for person, day, and their combination. Trait empathy = all trait empathy predictors. Valence and empathy adds valence to trait empathy as a main effect (in parentheses) and when testing for interactions. Emotion and empathy is the same for discrete emotions. Final two columns include only valence or only emotion as fixed effects.

Additional model performance statistics can be found in Table S3.

**Table S3**
Model Comparisons for Predictors Across Different Empathy Measures

| **Model Name** | **AIC (weights)** | **AICc (weights)** | **BIC (weights)** | ***R²* (cond.)** | ***R²* (marg.)** | **ICC** | **RMSE** | **Sigma** |
| --- | --- | --- | --- | --- | --- | --- | --- | --- |
| **Extent of Empathy Models** |  |  |  |  |  |  |  |  |
| Full Trait Empathy | 4027.6 (<.001) | 4027.9 (<.001) | 4093.3 (0.674) | 0.509 | 0.067 | 0.474 | 0.971 | 1.121 |
| Empathy Valence Main | 4018.9 (<.001) | 4019.3 (<.001) | 4094.7 (0.326) | 0.506 | 0.076 | 0.465 | 0.970 | 1.120 |
| Empathy Valence Interaction | 4010.8 (0.002) | 4012.8 (0.001) | 4177.6 (<.001) | 0.519 | 0.103 | 0.464 | 0.954 | 1.109 |
| Empathy Emotion Main | 3998.1 (0.998) | 3999.1 (0.999) | 4119.4 (<.001) | 0.529 | 0.098 | 0.478 | 0.943 | 1.099 |
| Empathy Emotion Interaction | 4038.9 (<.001) | 4068.4 (<.001) | 4660.5 (<.001) | 0.577 | 0.174 | 0.488 | 0.862 | 1.064 |
| Valence in Isolation | 4188.6 (<.001) | 4188.6 (<.001) | 4219.1 (<.001) | 0.498 | 0.009 | 0.493 | 0.964 | 1.115 |
| Emotion in Isolation | 4228.2 (<.001) | 4228.6 (<.001) | 4304.8 (<.001) | 0.504 | 0.030 | 0.488 | 0.964 | 1.116 |
| **Emotion Sharing Models** |  |  |  |  |  |  |  |  |
| Full Trait Empathy | 4995.9 (<.001) | 4996.2 (<.001) | 5061.7 (<.001) | 0.376 | 0.030 | 0.357 | 1.602 | 1.774 |
| Empathy Emotion Interaction | 4926.6 (<.001) | 4956.2 (<.001) | 5548.1 (<.001) | 0.450 | 0.177 | 0.332 | 1.436 | 1.686 |
| Empathy Emotion Main | 4874.2 (0.606) | 4875.2 (0.711) | 4995.4 (<.001) | 0.396 | 0.092 | 0.335 | 1.575 | 1.736 |
| Empathy Valence Interaction | 4875.0 (0.394) | 4877.0 (0.289) | 5041.8 (<.001) | 0.417 | 0.108 | 0.347 | 1.524 | 1.707 |
| Empathy Valence Main | 4895.7 (<.001) | 4896.1 (<.001) | 4971.5 (>.999) | 0.382 | 0.067 | 0.337 | 1.580 | 1.746 |
| Valence in Isolation | 5101.3 (<.001) | 5101.3 (<.001) | 5131.8 (<.001) | 0.373 | 0.036 | 0.349 | 1.579 | 1.746 |
| Emotion in Isolation | 5137.9 (<.001) | 5138.3 (<.001) | 5214.5 (<.001) | 0.378 | 0.063 | 0.336 | 1.596 | 1.753 |
| **Personal Distress Models** |  |  |  |  |  |  |  |  |
| Full Trait Empathy | 4812.7 (<.001) | 4837.9 (<.001) | 5451.9 (<.001) | 0.626 | 0.388 | 0.389 | 1.039 | 1.210 |
| Empathy Emotion Interaction | 4774.0 (>.999) | 4775.0 (>.999) | 4898.5 (<.001) | 0.589 | 0.334 | 0.382 | 1.105 | 1.246 |
| Empathy Emotion Main | 5252.6 (<.001) | 5252.8 (<.001) | 5320.0 (<.001) | 0.389 | 0.065 | 0.347 | 1.376 | 1.525 |
| Empathy Valence Interaction | 4796.3 (<.001) | 4798.1 (<.001) | 4967.5 (<.001) | 0.600 | 0.332 | 0.400 | 1.093 | 1.242 |
| Empathy Valence Main | 4796.4 (<.001) | 4796.8 (<.001) | 4874.2 (>.999) | 0.593 | 0.328 | 0.394 | 1.113 | 1.254 |
| Valence in Isolation | 5022.5 (<.001) | 5022.5 (<.001) | 5053.8 (<.001) | 0.584 | 0.274 | 0.427 | 1.124 | 1.266 |
| Emotion in Isolation | 5048.8 (<.001) | 5049.2 (<.001) | 5127.4 (<.001) | 0.586 | 0.284 | 0.421 | 1.113 | 1.257 |
| **Compassion Models** |  |  |  |  |  |  |  |  |
| Full Trait Empathy | 4372.0 (<.001) | 4372.3 (<.001) | 4437.8 (<.001) | 0.426 | 0.071 | 0.382 | 1.208 | 1.344 |
| Empathy Emotion Interaction | 4307.2 (>.999) | 4308.2 (>.999) | 4428.5 (<.001) | 0.451 | 0.103 | 0.388 | 1.178 | 1.322 |
| Empathy Emotion Main | 4372.2 (<.001) | 4401.7 (<.001) | 4993.8 (<.001) | 0.472 | 0.169 | 0.364 | 1.146 | 1.330 |
| Empathy Valence Interaction | 4334.7 (<.001) | 4335.1 (<.001) | 4410.5 (>.999) | 0.426 | 0.076 | 0.379 | 1.210 | 1.348 |
| Empathy Valence Main | 4349.0 (<.001) | 4351.0 (<.001) | 4515.8 (<.001) | 0.447 | 0.086 | 0.395 | 1.185 | 1.336 |
| Valence in Isolation | 4510.1 (<.001) | 4510.2 (<.001) | 4540.7 (<.001) | 0.426 | 0.005 | 0.423 | 1.188 | 1.331 |
| Emotion in Isolation | 4519.7 (<.001) | 4520.1 (<.001) | 4596.2 (<.001) | 0.448 | 0.031 | 0.430 | 1.156 | 1.304 |
| **Efficacy Models** |  |  |  |  |  |  |  |  |
| Full Trait Empathy | 2613.0 (<.001) | 2613.3 (<.001) | 2678.8 (<.001) | 0.583 | 0.149 | 0.510 | 0.545 | 0.615 |
| Empathy Emotion Interaction | 2636.6 (<.001) | 2666.0 (<.001) | 3258.6 (<.001) | 0.618 | 0.221 | 0.510 | 0.524 | 0.608 |
| Empathy Emotion Main | 2574.9 (<.001) | 2575.9 (<.001) | 2696.3 (<.001) | 0.612 | 0.169 | 0.533 | 0.529 | 0.599 |
| Empathy Valence Interaction | 2521.4 (0.003) | 2523.4 (0.002) | 2687.9 (<.001) | 0.625 | 0.189 | 0.537 | 0.518 | 0.589 |
| Empathy Valence Main | 2510.1 (0.997) | 2510.5 (0.998) | 2585.8 (>.999) | 0.620 | 0.182 | 0.535 | 0.524 | 0.591 |
| Valence in Isolation | 2654.0 (<.001) | 2654.0 (<.001) | 2684.5 (<.001) | 0.618 | 0.032 | 0.605 | 0.518 | 0.589 |
| Emotion in Isolation | 2713.6 (<.001) | 2714.0 (<.001) | 2790.1 (<.001) | 0.612 | 0.032 | 0.600 | 0.522 | 0.596 |

*Note.*  Model statistics calculated using the compare performance function from the performance package in R.

**
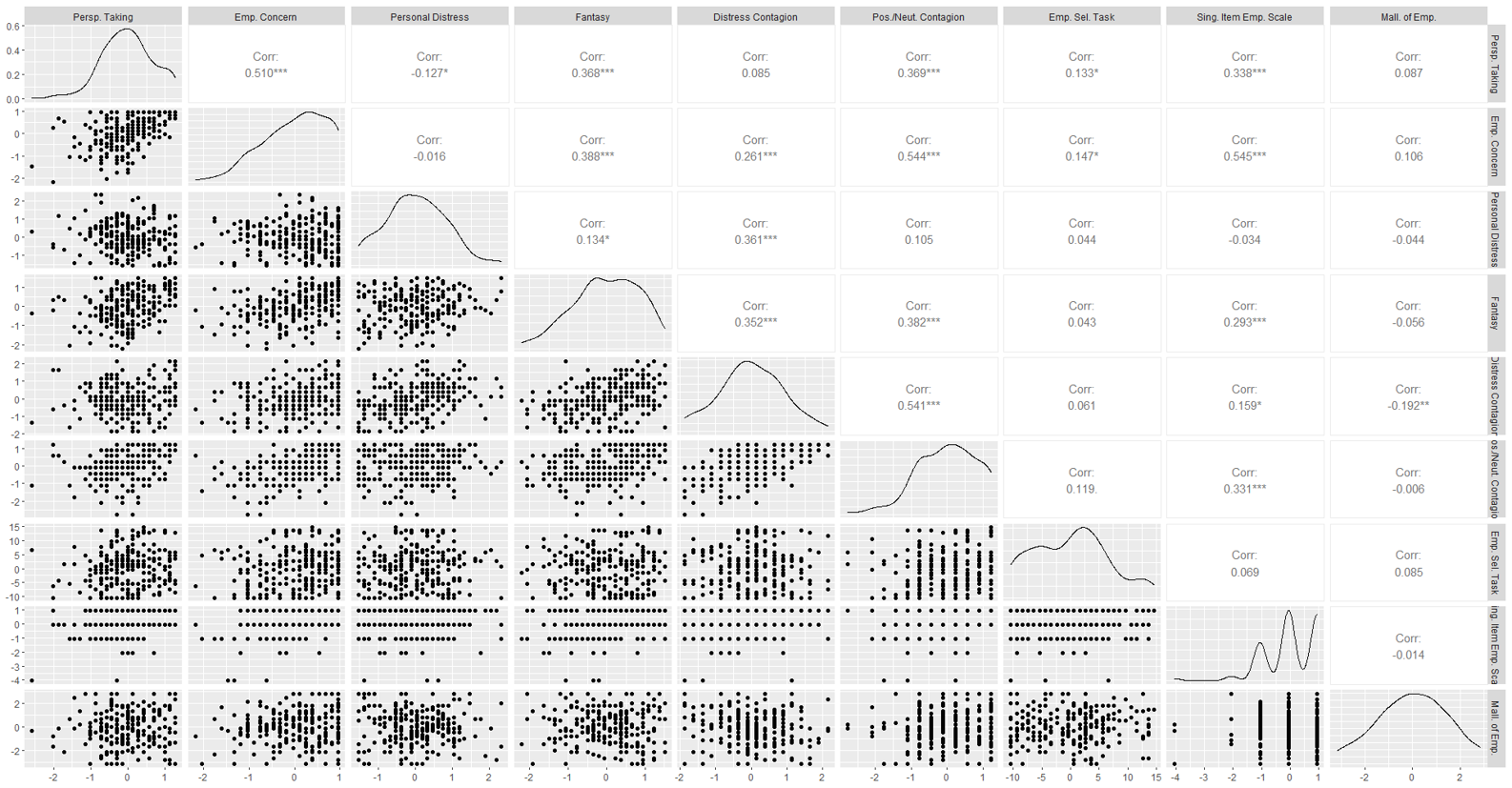
**

**Figure S1.** Correlations between trait empathy measures. First, we have the perspective taking, empathic concern, personal distress, and fantasy subscales of the Interpersonal Reactivity Index (IRI). Next, we have the distress contagion and positive neutral contagion subscales of the Empathy Index (EI). Finally, we have the empathy selection task (EST), single item trait empathy scale (SITES), and the beliefs about malleability of empathy scale.


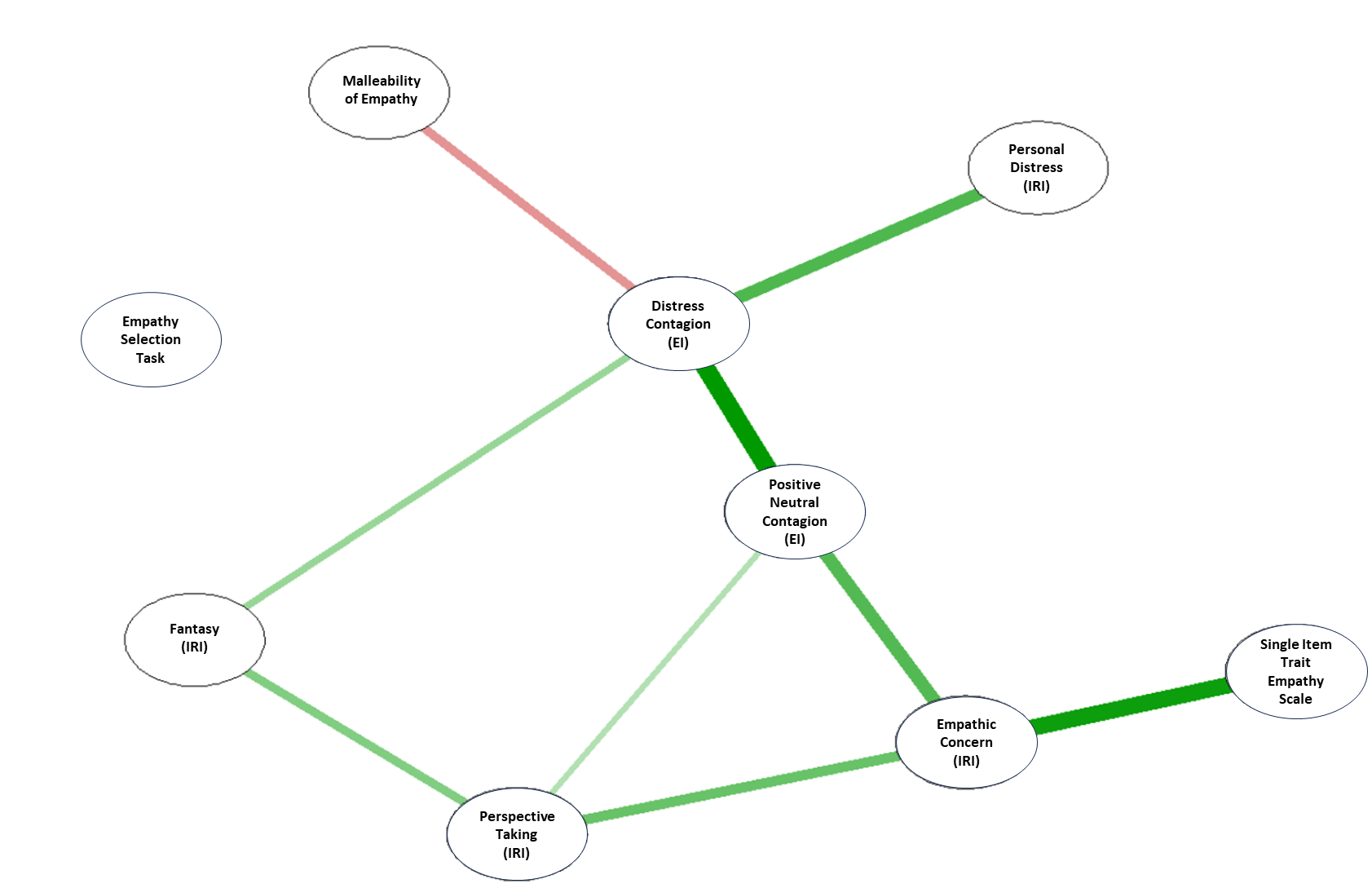


**Figure S2.** Autoregressive correlation network graph of trait empathy variables. Plots a Pairwise Randomized Markov Field (PRMF) of correlations accounting for all other correlations in the network. Variables are represented by nodes and correlations are illustrated with connecting edges which are thicker for stronger correlations and colored green for positive and red for negative.

Weight matrix for the network graph is provided below.

IRI_E IRI_PT IRI_PD IRI_F EI_D EI_P SIT M_ EST

IRI_E 0.00 0.27 0.00 0.00 0.00 0.30 0.43 0.00 0

IRI_PT 0.27 0.00 0.00 0.22 0.00 0.14 0.00 0.00 0

IRI_PD 0.00 0.00 0.00 0.00 0.31 0.00 0.00 0.00 0

IRI_F 0.00 0.22 0.00 0.00 0.18 0.00 0.00 0.00 0

EI_D 0.00 0.00 0.31 0.18 0.00 0.45 0.00 -0.19 0

EI_P 0.30 0.14 0.00 0.00 0.45 0.00 0.00 0.00 0

SIT 0.43 0.00 0.00 0.00 0.00 0.00 0.00 0.00 0

M__ 0.00 0.00 0.00 0.00 -0.19 0.00 0.00 0.00 0

EST 0.00 0.00 0.00 0.00 0.00 0.00 0.00 0.00 0

**Table S4.** Participant centered correlations of state empathy

**
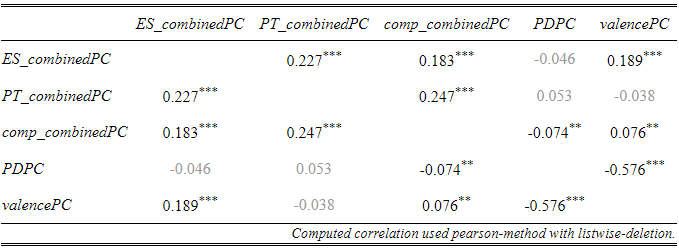
**

*Note.* Shows deviations from one’s own average on different state empathy components (and valence) correlate with each other. For example, when participants feel compassion more than their own average, they also perspective take and share emotions more than their own average but experience lower than average personal distress. Observing more positive than average emotions predicts greater emotion sharing and compassion but much lower personal distress.

**Table S5.** Correlations between trait empathy and participant mean state empathy

**Trait State r**

Perspective Taking (IRI) State Perspective Taking 0.27

Empathic Concern (IRI) State Compassion 0.28

Fantasy (IRI) State Empathy Opportunities 0.20

Distress Contagion (EI) State Personal Distress 0.21

Positive/Neutral Contagion (EI) State Emotion Sharing 0.21

Single Item Trait Empathy Scale Extent of State Empathy 0.31

*Note.* Shows how select trait empathy measures correlate with participant mean state empathy. While trait to single state correlations were similar, the trait to mean state empathy correlations are lower than Big Five trait to mean state correlations, extraversion: .38, conscientiousness: .44, emotional stability: .49, agreeableness: .50, and intellect: .56.

Results from non-winning models are presented for exploratory purposes here.

At the emotion level, individuals were more inclined to share happiness, b = 1.20, SE = 0.28, t(1121) = 4.27, p < .001, r = 0.13, and humour, b = 0.80, SE = 0.34, t(1109) = 2.39, p = .017, r = 0.07, relative to pain.


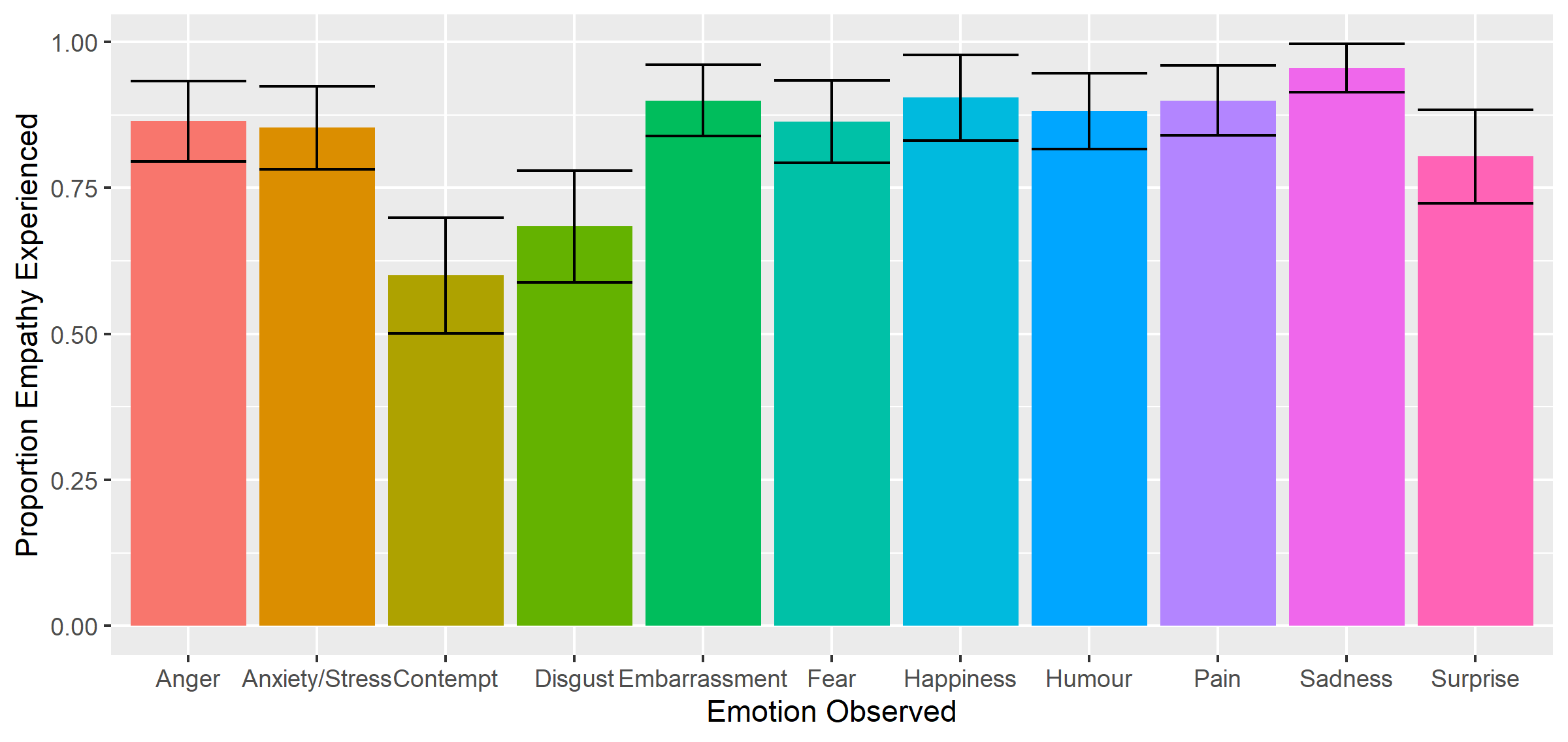


**Figure S3. Proportion of empathy opportunities that empathy was actually experienced for different target emotions.** Empathy was reported at high rates overall for most emotions but was reported at significantly lower rates when observing contempt and disgust. This model was not ultimately selected as the best model and results presented here are exploratory and require confirmation in future work.


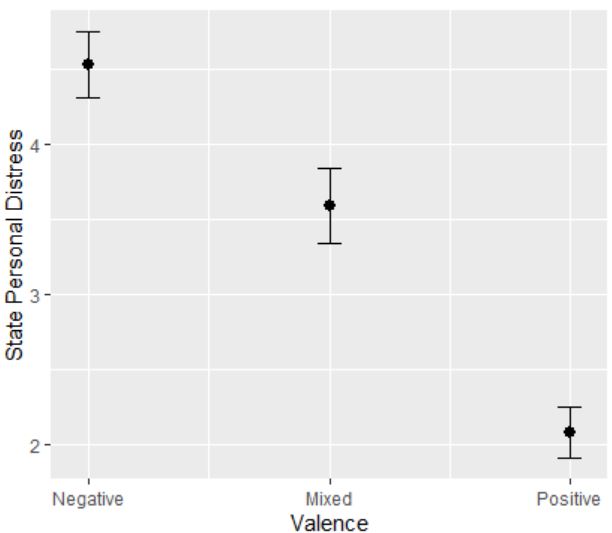
**
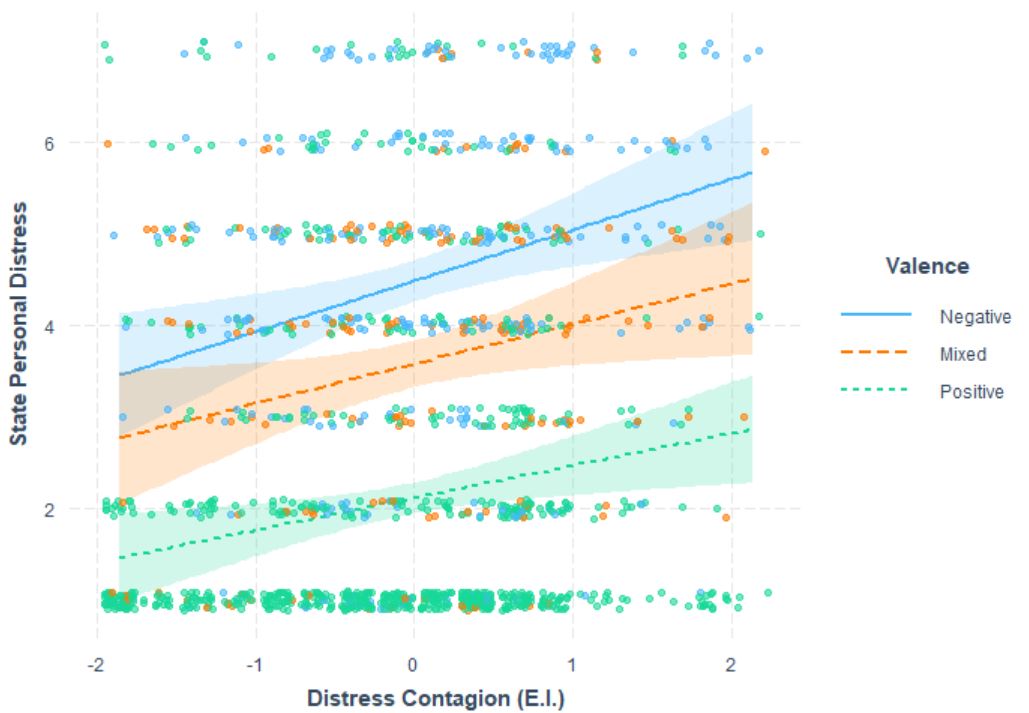
**

**
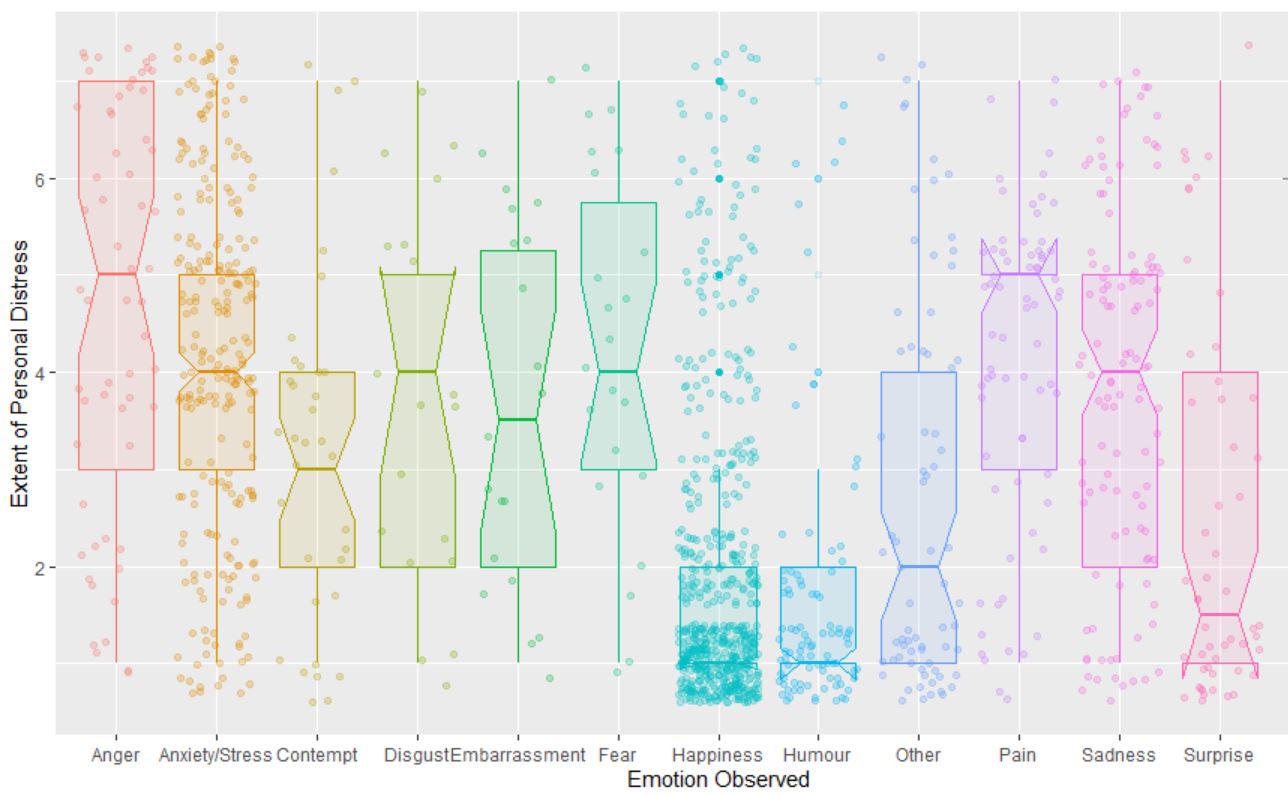
**

**Figure S4.** State personal distress was lower during mixed emotions and very low during positive emotions (top left). Trait distress contagion was associated with increased personal distress in everyday life across valence including positive, b = 0.35, SE = 0.13, p = .01, mixed, b = 0.43, SE = 0.19, p = .02, and negative, b = 0.55, SE = 0.17, p < .001, emotions (top right). Experiences of personal distress in everyday life varied considerably across different emotions. Dots are personal distress scores from individual survey where a specific emotion was reported, jittered to show frequency. Lines indicate the median, and notches show 95% confidence intervals for the median. Anger and pain showed the highest distress, this was followed by a second tier of anxiety/stress, disgust, fear, and sadness (bottom).
